# Accuracy of fruit-fly eclosion rhythms evolves by strengthening circadian gating rather than developmental fine-tuning

**DOI:** 10.1242/bio.042176

**Published:** 2019-08-15

**Authors:** Vishwanath Varma, Shambhavi Krishna, Manishi Srivastava, Vijay Kumar Sharma, Vasu Sheeba

**Affiliations:** 1 Chronobiology Laboratory, Evolutionary and Integrative Biology Unit, Jawaharlal Nehru Centre for Advanced Scientific Research, Jakkur, Bangalore 560064, Karnataka, India; 2 School of Natural Sciences and Engineering, Animal Behaviour and Cognition Programme, National Institute of Advanced Studies, Indian Institute of Science Campus, Bangalore 560012, Karnataka, India; 3 Behavioural Neurogenetics Laboratory, Neuroscience Unit, Jawaharlal Nehru Centre for Advanced Scientific Research, Jakkur, Bangalore 560064, Karnataka, India

**Keywords:** Development, Circadian clocks, Drosophila, Gating, Eclosion, Evolution, Accuracy

## Abstract

Fruit flies (*Drosophila melanogaster*) eclose from their pupae mainly around dawn. The timing of eclosion is thought to confer adaptive benefits to the organisms and thus shows remarkable accuracy. However, it is not clear what factors are involved in the evolution of such accuracy in natural populations. In this study, we examined the relative contributions of gating of eclosion by the circadian clock versus clock-independent developmental rates and light-induced responses in the eclosion phenotype exhibited by fly populations that have evolved greater accuracy in eclosion rhythms compared to controls. We compared variation in timing of transitions between early developmental stages (pupariation and pigmentation), overall development time under constant light conditions – where circadian clocks are dysfunctional – and eclosion profiles when developmental rate was manipulated using different larval densities in selected and control stocks. Our results showed that stocks that have evolved greater accuracy of eclosion rhythms due to artificial selection do not show reduced individual variation in pupariation and pigmentation time compared to controls, though they do exhibit lower variation in eclosion time. Selected stocks also did not show lower variation in eclosion time under constant light conditions in contrast to the greater accuracy seen under light-dark cycles. Moreover, manipulations of developmental rate by varying larval density and inducing eclosion by changing onset of light phase did not alter the eclosion profile of selected stocks as much as it did controls, since selected stocks largely restricted eclosion to the daytime. These results suggest that fly populations selected for greater accuracy have evolved accurate eclosion rhythms primarily by strengthening circadian gating of eclosion rather than due to fine-tuning of clock-independent developmental processes.

This article has an associated First Person interview with the first author of the paper.

## Abstract (precis)

**Summary:** Fruit fly populations that have evolved greater accuracy of eclosion rhythms show enhanced gating of their eclosion by circadian clocks but not reduced variation in clock-independent developmental processes.

## INTRODUCTION

Eclosion rhythms are thought to have evolved as a consequence of adaptive benefits conferred to insects by emerging in the early part of the day (Pittendrigh, 1954; Cloudsley-Thompson, 1960; Tanaka and Watari, 2009). If so, it would be necessary to effectively restrict the timing of eclosion in order to maximize these benefits. The daily timing of eclosion (or eclosion phase) in *Drosophila melanogaster* populations is noted to be highly accurate (Kannan et al., 2012a) though it is not clear how such accuracy evolves and what factors may contribute to accuracy of the eclosion rhythm.

Rhythmic behaviours such as eclosion and locomotor activity in *D. melanogaster* are known to be under the control of the circadian clock. The circadian clock is an internal time-keeper constituted by transcription-translation feedback loops involving several clock proteins which sustain roughly 24-h molecular rhythms (Dunlap, 1999; Reppert and Weaver, 2002). Accuracy of circadian phase or maintaining a stable phase relationship of circadian rhythms with respect to the external environment is recognized as an important function of the circadian clock (Pittendrigh, 1981). Theoretical studies on accuracy and internal periodicity and clock-resetting have suggested a close relationship between the accuracy of phase and these clock properties (Pittendrigh and Daan, 1976a; Beersma et al., 1999). However, factors independent of the circadian clock may also affect the accuracy of circadian phase of behavioural output. For instance, Pittendrigh and Daan (1976b) noted that the core circadian clock appeared to be more precise than the overt rhythm and suggested that variability in output processes is a significant component of variability in phase of the rhythm. Hence, accuracy of circadian behaviours could possibly evolve by reducing variability of such output processes. Further, studies from intertidal midges have suggested that addition of a proximate cue can improve synchronization in eclosion timing (Soong et al., 2006). Therefore, increased sensitivity to proximate cues can potentially enhance accuracy of circadian rhythms as well. However, there are no reports of comprehensive studies examining the relative roles of clock-dependent and clock-independent factors in the evolution of accurate circadian rhythms.

Eclosion in fruit flies is a widely studied rhythm, the timing of which is determined by developmental state and circadian gating (Qiu and Hardin, 1996; Myers, 2003). While eclosion is the culmination of development through larval and pupal stages, which is a sequential progression of developmental stages involving predominantly clock-independent mechanisms, the final act of ecdysis (or eclosion) is controlled by both circadian clock gating as well as several neuro-endocrine processes exclusively seen at this stage. Although transition to early developmental stages such as pupariation and pigmentation are known to be gated by the circadian clock in many species of insects such as *Anopheles gambiae* and *Triatoma infestans* (Truman, 1972; Jones and Reiter, 1975; Fujishita and Ishizaki, 1982), pupariation and pigmentation in *D. melanogaster* do not show circadian rhythmicity (Pittendrigh and Skopik, 1970; Qiu and Hardin, 1996). However, the timing of pupariation and pigmentation is an important determinant of the timing of eclosion (Pittendrigh and Skopik, 1970; Qiu and Hardin, 1996). For instance, most flies showing wing pigmentation before the end of the light phase eclosed between ZT2 and ZT12 on the next day (where ZT0 or Zeitgeber Time 0 is the time of lights-on and ZT12 is the time of lights-off under a 12:12 h light-dark cycle or LD 12:12), while those showing pigmentation during the dark phase eclosed before ZT2 on the day following the subsequent light phase (Qiu and Hardin, 1996). Hence, timing of eclosion and its accuracy may be modulated in *Drosophila* populations by altering the timing of preceding developmental stages such as wing pigmentation. Moreover, there is known to be significant inter-individual variation in developmental rates even in developmentally synchronous populations reared at constant temperature (Pittendrigh and Skopik, 1970). Additionally, the proximate mechanisms leading up to the final stage of eclosion (or ecdysis) may also show high levels of variation between individuals (Pittendrigh and Skopik, 1970). These developmental processes are partly independent of the circadian clock and the gating of eclosion imposed by entrainment to LD cycles. Hence, examining the overall development time (from egg collection to eclosion) under constant conditions and comparing it with the development time under LD cycles may be a useful approach to distinguish between developmental processes independent of the circadian clock and effects of gating by the clock.

Nevertheless, inferences regarding the role of developmental processes in determining the timing of eclosion under an external cycle may be inaccurate if these are based solely upon independent assessments of their roles under constant environments since several physiological processes interact among each other and with the environment at various stages of development. Developmental rate itself is affected by larval density and internal clock period (Peters and Barbosa, 1977; Kyriacou et al., 1990) in addition to light and temperature (Bonnier, 1926; Paranjpe et al., 2005). Although circadian clocks appear to be operating early during development (Sehgal et al., 1992) and pigment-dispersing factor (PDF)-secreting circadian clock neurons appear in early larval stages (Helfrich-Förster, 1997), they assess developmental state for competence to eclose in the next available gate only around the time of wing pigmentation (Qiu and Hardin, 1996). Since clock neurons project to neurons involved in prothoracicotrophic hormone (PTTH) secretion (ŽITňAN et al., 1993) as well as neurons that synapse onto the prothoracic gland (PG; Siegmund and Korge, 2001) which are involved in assessment of growth (Mirth et al., 2005), the gating of eclosion by the circadian clock may occur by regulation of ecdysteroid production from the PG. Recent studies have implicated short neuropeptide F (sNPF) in transmitting clock information from clock neurons to the PTTH neurons which in turn contact the PG (Selcho et al., 2017). Ecdysteroid production induced by PTTH follows the decline of juvenile hormone (JH) levels and decreasing ecdysteroid level is an important cue for the initiation of eclosion behaviours in *Manduca sexta* (Pelc and Steel, 1997; Truman, 1983). These eclosion behaviours are triggered by a hormonal cascade involving pre-ecdysis-triggering hormone (PETH), ecdysis-triggering hormone (ETH), eclosion hormone (EH) and crustacean cardioactive peptide (CCAP; Zitnan et al., 1996, 1999; Ewer et al., 1997; Gammie and Truman, 1997). The release of CCAP triggered by EH is thought to be the final step in the ecdysis pathway, which induces the ecdysis motor program (Gammie and Truman, 1997). While expression of LARK protein, which is known to regulate neuronal excitability in CCAP neurons (Huang et al., 2007), exhibits circadian control and may regulate circadian gating of adult eclosion (McNeil et al., 1998; Zhang et al., 2000), the circuit connecting these cells to clock neurons remains uncharacterized. Thus, circadian clocks interact with neuroendocrine pathways and the environmental cycles at multiple levels and stages of development. Hence, manipulations of developmental rate under external cycles may reveal greater insight into the relative influences of clock-dependent and independent processes on eclosion time under such conditions.

In addition to these neuropeptidergic signals and developmental factors, eclosion can be directly induced by light input via the compound eyes and ocelli (McNabb and Truman, 2008). The lights-on signal is thought to induce eclosion by stimulating release of EH or suppressing the inhibition of eclosion following EH release (McNabb and Truman, 2008). Thus, direct responses or masking effects of light can also help in determining the timing of eclosion in flies and can possibly reduce variation in eclosion timing since a lights-on cue can induce large numbers of flies to emerge simultaneously, like the phenomenon observed in inter-tidal midges (Soong et al., 2006).

In this study, we examined the contributions of such non-clock processes involved in the regulation of eclosion time in the evolution of greater accuracy in fruit fly populations selected for narrow gate of eclosion. Since multiple interactions between internal physiology and ecological variables could affect the fitness of eclosion behaviour at a particular time of the day, we wished to determine the relative roles of clocks and developmental mechanisms in the responses to possible selection pressures in the environment. The selected populations in our study had evolved significantly greater eclosion in the selection window (ZT1–ZT2; Kannan et al., 2012a) as well as lower day-to-day variability or enhanced accuracy in the timing of the peak of eclosion (Kannan et al., 2012a). However, the differences in accuracy of activity-rest rhythm between selected and control populations are much smaller and less robust to changes in environmental conditions (Kannan et al., 2012b). Although some properties of core circadian clocks have been altered as a consequence of selection (Kannan et al., 2012a), these are insufficient to explain the magnitude of increase in accuracy of eclosion rhythms in selected populations compared to controls. Moreover, developmental processes could have been altered as an indirect response to selection as previous studies have reported links between the circadian system and development (Nikhil et al., 2016; Yadav and Sharma, 2013). Since clock-independent downstream processes contribute disproportionately to variability in overt rhythms (Pittendrigh and Daan, 1976a) and masking responses to external cues can also increase accuracy of rhythms with respect to an external cycle, we hypothesized that early developmental durations and masking of eclosion to light may have also evolved in these populations in order to enhance the accuracy of eclosion rhythms. Hence, we examined various aspects of developmental processes such as timing of transition between developmental stages (egg-to-adult development preceding the final act of eclosion) and effects of manipulation of developmental rate by varying larval density under LD cycles, development time under constant external environments, and masking effects of light on eclosion timing in selected and control stocks. Reduction in variability in the timing of early developmental stages such as pupariation and pigmentation which are not known to be under circadian control may directly result in reduced variability in eclosion timing. The influence of developmental rates on the eclosion profile of the two stocks can be further examined by assaying eclosion rhythms of both stocks under lower larval densities where development is faster. Furthermore, clock-independent processes leading up to the final stage of eclosion may have innately lower variability in the selected stocks. This can be tested under constant light (LL) conditions where the role of clock is absent. Finally, accuracy may be improved simply by enhancing sensitivity to a proximate cue such as light, which we tested by assaying the masking response of selected and control stocks to light. Our results did not reveal any reduction of inter-individual variation in timing of pupariation or pigmentation. Furthermore, variation in eclosion time under constant conditions was not lower in selected stocks compared to controls, suggesting the necessity of circadian gating for higher accuracy of selected stocks. Moreover, selected stocks continued to eclose well within their narrow circadian gate even when developmental rate was manipulated by assaying eclosion at different larval densities. These stocks also did not show significant differences in masking responses to light cues with respect to controls though they continued to restrict their eclosion to the duration of the circadian gate more effectively than controls under all circumstances. These results suggest that selected stocks have evolved greater accuracy of eclosion rhythms primarily due to enhanced gating of eclosion by the circadian clock and not due to accumulated reduction in variation at previous stages of development or differences in masking responses between the stocks.

## RESULTS

### Pupariation, pigmentation and eclosion of selected and control stocks under LD 12:12

**Fig. 1.**
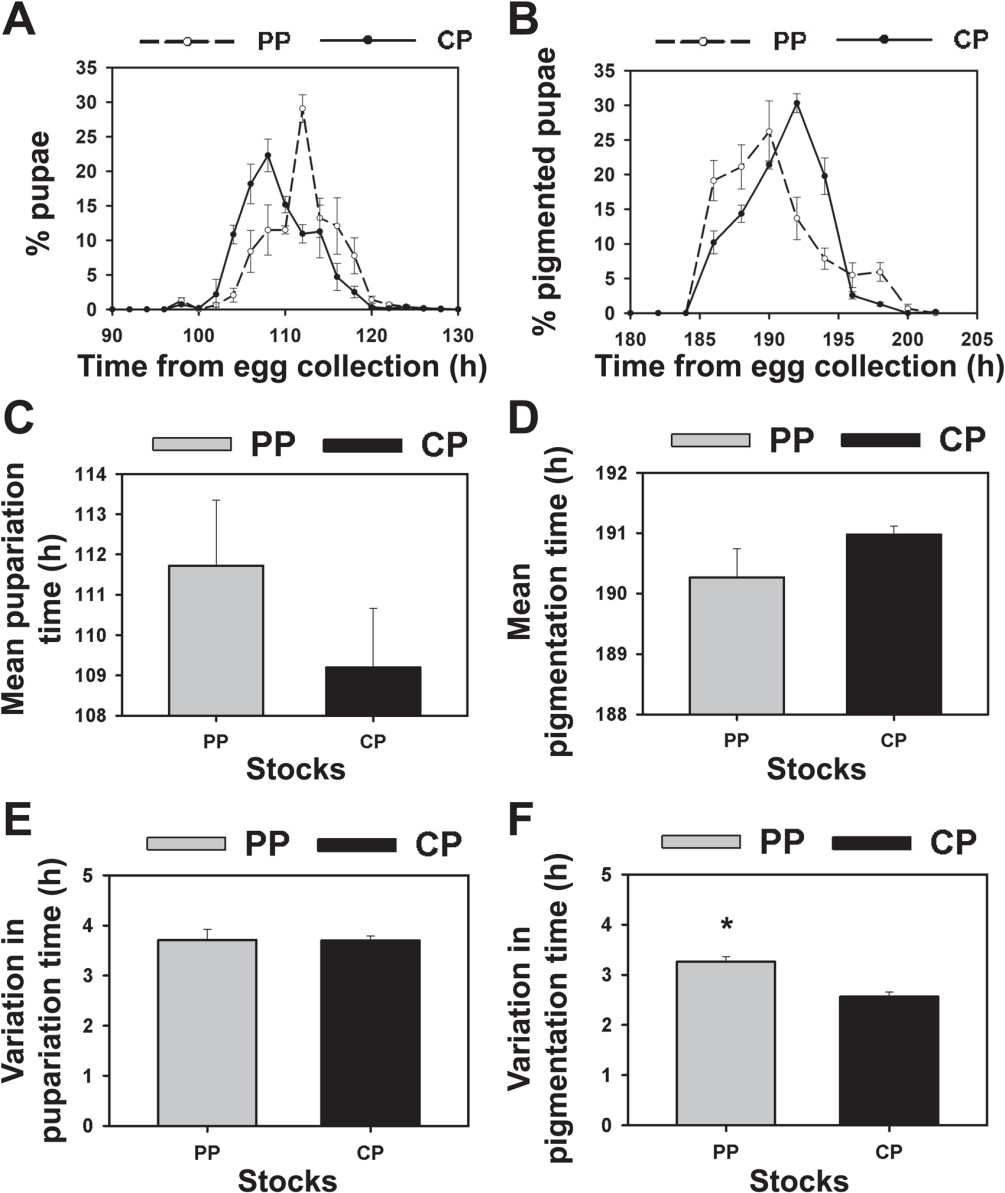
**Pupariation and pigmentation in selected and control populations under LD 12:12.** (A) Percentage of larvae that pupated in every 2 h interval measured from the time of egg collection in selected (PP, total *n*=1085 pupae across four populations) and control (CP, total *n*=1134) stocks under LD 12:12. (B) Percentage of pupae from selected (*n*=1084 pupae) and control (*n*=1119) stocks that showed wing pigmentation in every 2 h interval under LD 12:12. (C) Mean pupariation time of selected and control stocks under LD 12:12. (D) Mean pigmentation time of selected and control stocks under LD 12:12. (E) Variation in pupariation time estimated by the standard deviation in pupariation time across all individuals of selected and control stocks. (F) Variation in pigmentation time across all individuals of selected stocks is greater than that of control stocks (*P*<0.05). All values are estimated from single vials containing 30 flies and subsequently averaged across 10 vials for each replicate population. Bar graphs and line plots represent mean values and error bars are s.e.m. across four replicate populations for each stock (*n*=4). All statistically significant differences reported are based on ANOVA followed by post-hoc comparisons using Tukey's HSD test. *P<0.05.

We assayed the timing of transitions between developmental stages such as pupariation and wing pigmentation that are not known to be under clock control in *Drosophila* to determine whether the selected stocks have evolved differences in developmental rate (Fig. 1A–F). We found no significant change in the pupariation profile in the selected stocks compared to control stocks (Fig. 1A). Whereas maximum pupariation was seen in control stocks at ∼106 h after egg-collection, pupariation peak was seen at ∼112 h for the selected stocks (Fig. 1A). However, the mean pupariation time of selected and control stocks and their standard deviation were not significantly different from each other (*P*>0.05; Fig. 1C,E; Table 1). Since we found marginal differences in the pupariation profile between selected and control stocks, we looked at the profiles of replicate populations but found large variation across these populations which were not consistent within the stocks (Fig. S1). Moreover, we also did not find significant differences in pupariation time between the stocks when this experiment was repeated (Fig. S2). These results suggest that pupariation profiles of selected and control stocks are largely similar and the mean pupariation time and variation between individuals are not significantly different between selected and control stocks (Fig. 1A,C,E).

**Table 1.**
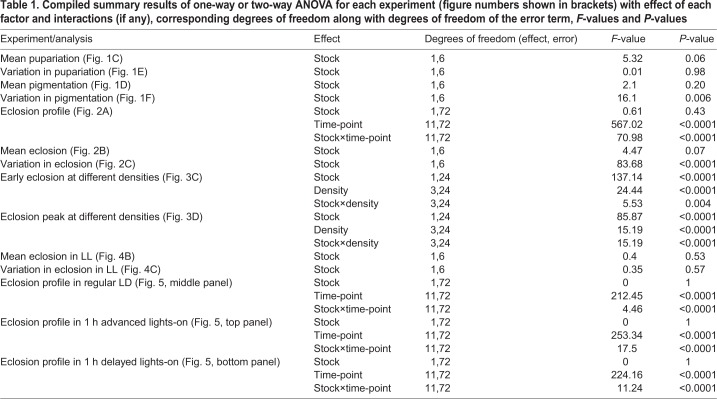
**Compiled summary results of one-way or two-way ANOVA for each experiment (figure numbers shown in brackets) with effect of each factor and interactions (if any), corresponding degrees of freedom along with degrees of freedom of the error term, *F*-values and *P*-values**

We also recorded the time that these pupae started showing wing pigmentation and found that the timing of wing pigmentation was largely similar in the selected and control stocks. The peak of wing pigmentation was at ∼190 h after egg-collection in the selected stocks while the peak was at ∼192 h in the controls (Fig. 1B). These results suggest that wing pigmentation time is marginally advanced in the selected stocks with respect to the controls though not statistically significant (*P*>0.05; Fig. 1D; Table 1). However, the selected stocks showed significantly higher standard deviation in pigmentation time compared to control stocks (*P*<0.05; Fig. 1D; Table 1). Hence, it appears that the selected stocks are not different in their mean pigmentation time but show greater inter-individual variation in pigmentation time compared to control stocks (Fig. 1D,F). Thus, these differences do not explain the accuracy of eclosion rhythms seen in the selected stocks.

**Fig. 2.**
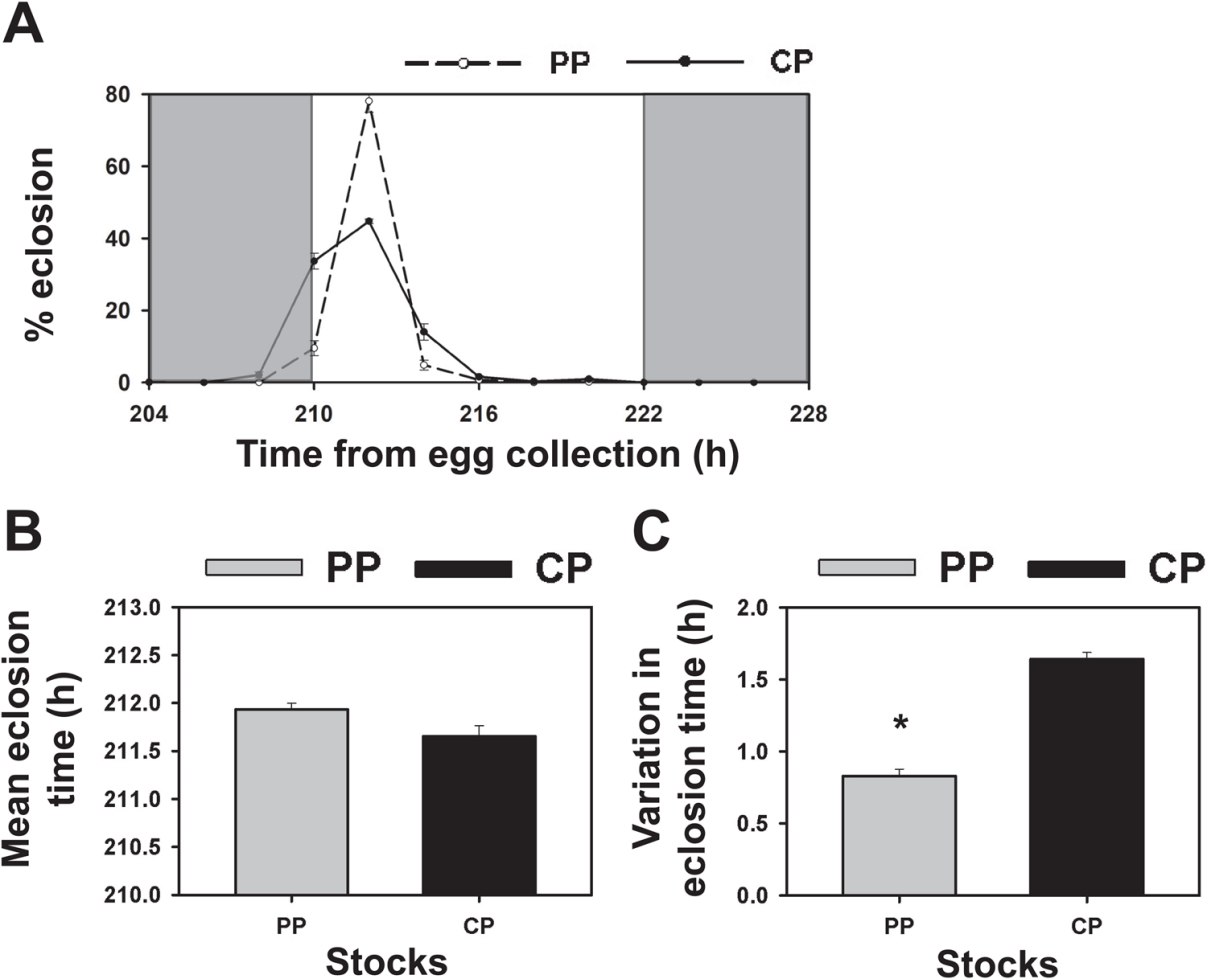
**Eclosion in selected and control populations under LD 12:12.** (A) Percentage of adult flies that eclosed in every 2 h interval measured from the time of egg collection in selected (PP, *n*=1044 flies) and control (CP, *n*=1083) stocks under LD 12:12. Grey shading represents dark phase. (B) Mean eclosion time of selected and control stocks under LD 12:12. (C) Variation in eclosion time estimated by the standard deviation in eclosion time across all individuals of selected stocks is lower than that of control stocks eclosing on second day of eclosion (*P<0.05). Error bars are s.e.m. across four replicate populations for each stock (*n*=4). Rest of the details as in Fig. 1.

We also assayed the timing of eclosion from the time of egg collection of both selected and control flies following pupariation and pigmentation described above to compare the differences in early development to the final stage of eclosion of the same set of flies. Since only 30 eggs were collected in each vial, eclosion occurred mainly on the 9th day with negligible eclosion (<5%) occurring on the 8th day and no eclosion after the 9th day. Hence, the figure depicts only eclosion on the 9th day (Fig. 2A). The eclosion of flies in the selected stocks peaked at ZT2 (which includes the selection window of ZT1–ZT2) as expected and the peak was sharp and narrow with very little eclosion before and after this peak (Fig. 2A). While control stocks also showed a peak of eclosion at ZT2, this peak was lower and their eclosion was more spread out compared to selected stocks with greater eclosion both prior to lights-on and after the peak at ZT2 (Fig. 2A). ANOVA followed by post-hoc comparisons revealed that eclosion at ZT2 was higher, and at ZT0 and ZT4 was lower, in the selected stocks compared to controls (Fig. 2A; *P*<0.05; Table 1). Thus, selected stocks do not show delay in eclosion time despite starting eclosion later since they also terminate eclosion earlier. This is also indicated by the lack of significant difference in mean eclosion time between selected and control stocks (*P*>0.05; Fig. 2B; Table 1) while standard deviation in eclosion time was significantly lower in the selected stocks compared to controls (*P*<0.05; Fig. 2C; Table 1). Hence, selected stocks show reduced variation in eclosion time even at low densities (30 eggs/vial) similar to the accuracy of eclosion previously reported at higher densities (Kannan et al., 2012a) compared to control stocks, despite no reduction in variation observed in pupariation or pigmentation time. Moreover, despite delay in pupariation time in selected stocks, they do not show overall delay in mean eclosion time compared to controls.

### Eclosion profiles of selected and control stocks at different larval densities

**Fig. 3.**
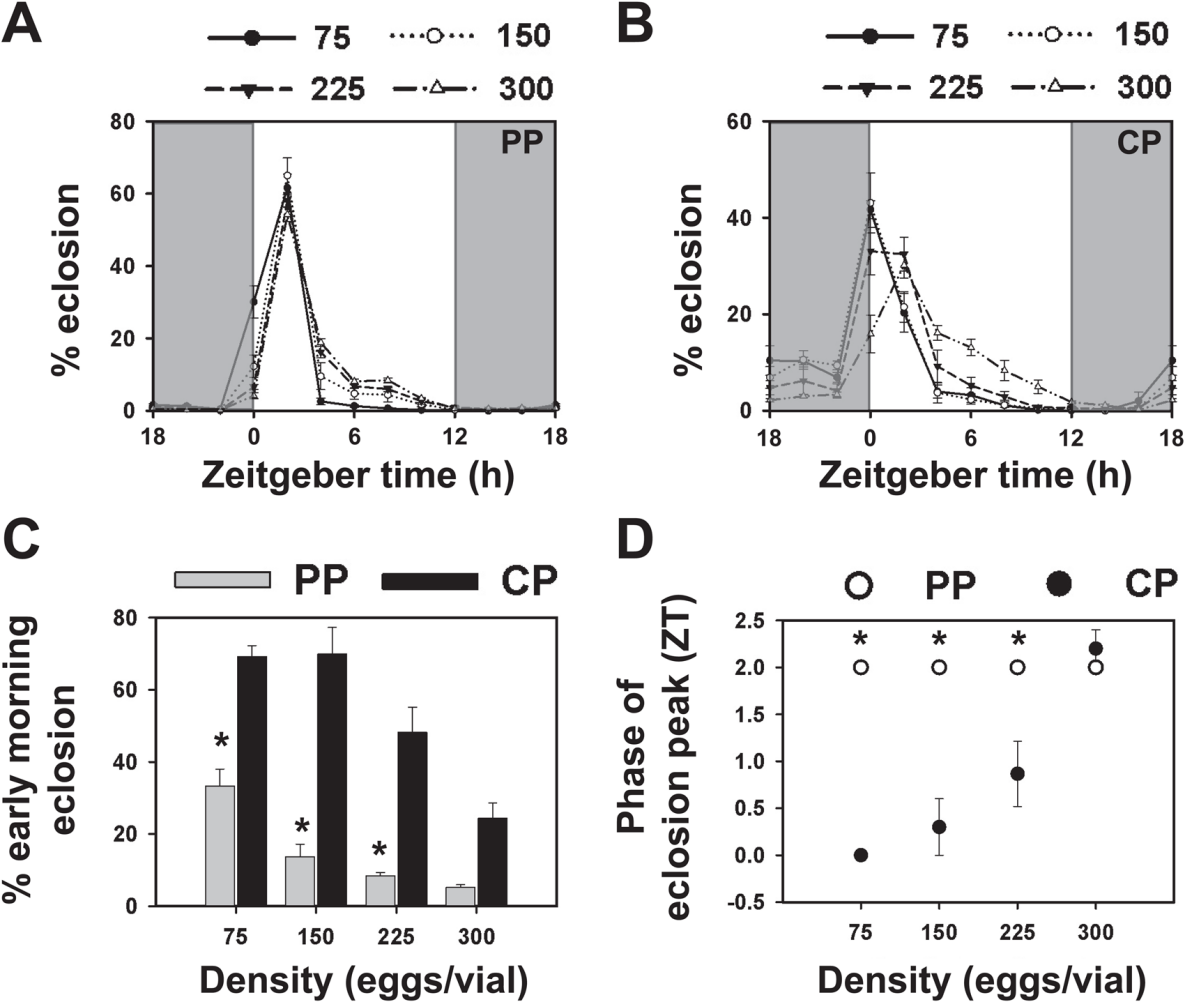
**Eclosion of selected (PP) and control (CP) stocks at different egg densities (eggs/vial) under LD 12:12.** (A) Eclosion profiles in 2 h intervals of selected stocks (total *n*=9603 flies across all densities) at 75, 150, 225 and 300 eggs/vial. Grey shading represents the dark phase. (B) Eclosion profiles of control stocks (*n*=7642 across all densities) at different egg densities. (C) Percentage eclosion in the early morning (or late night; ZT18–ZT0) as a fraction of total eclosion in the day. Black bars indicate selected stocks and grey bars indicate controls where selected stocks show lower early-morning eclosion compared to controls at 75, 150 and 225 eggs/vial (*P<0.05). (D) Phase of peak of eclosion (expressed in Zeitgeber Time) as a function of egg densities is significantly delayed in selected stocks (open circles) compared to control stocks (filled circles; *P<0.05). Error bars are s.e.m. across four replicate populations for each stock (*n*=4). Rest of the details same as in Fig. 1.

We also examined profiles of eclosion rhythms under different larval densities, where developmental rates increase with decreasing densities, to evaluate the role of clock-independent developmental rate on the eclosion rhythm of selected and control stocks. Fig. 3A represents average eclosion profiles of flies across 2–4 days (spanning across days 8–11), since vials with low densities show eclosion mainly on days 8 and 9 while eclosion occurs mainly on days 9–11 at higher densities. The eclosion prior to lights-on was greatly enhanced at lower densities in the control stocks such that eclosion peak shifts to ZT0 at lower densities while it remains at ZT2 in selected stocks irrespective of larval density (Fig. 3A,B). Although selected stocks also had greater eclosion at ZT0 at lower densities in contrast to the minimal eclosion at ZT0 at higher densities, there was still much lower eclosion seen before lights-on compared to the control stocks at low densities where developmental rate is increased (Fig. 3A), suggesting that reduced eclosion before lights-on is not due to longer development time in these stocks. ANOVA followed by post-hoc comparisons using Tukey's HSD revealed significantly higher early-morning eclosion in control stocks before lights-on at 75 and 150 eggs/vial compared to that seen at 225 and 300 eggs/vial (*P*<0.05; Fig. 3C; Table 1). However, only 75 eggs/vial density values were lower than that at 225 and 300 eggs/vial in the selected stocks (*P*<0.05; Fig. 3C; Table 1), suggesting that these stocks show lower responses to developmental rate manipulations than the controls. Moreover, the selected stocks showed lower eclosion before lights-on compared to control stocks at 75, 150 and 225 eggs/vial (*P*<0.05; Fig. 3C). Additionally, the peak of eclosion in selected stocks (which occurred at ZT2 at all densities) was significantly delayed compared to control stocks at 75, 150 and 225 eggs/vial (*P<*0.05; Fig. 3D). This shows that eclosion peak in selected stocks remains accurate across densities while eclosion peak of control stocks occurs earlier at lower densities compared to their corresponding peak at 300 eggs/vial (*P<*0.05; Fig. 3D; Table 1). Thus, the eclosion profiles and timing of eclosion peak of selected stocks are relatively more robust to changes in larval densities (or manipulations of developmental rate) as compared to controls.

### Eclosion of selected and control stocks under LL conditions

**Fig. 4.**
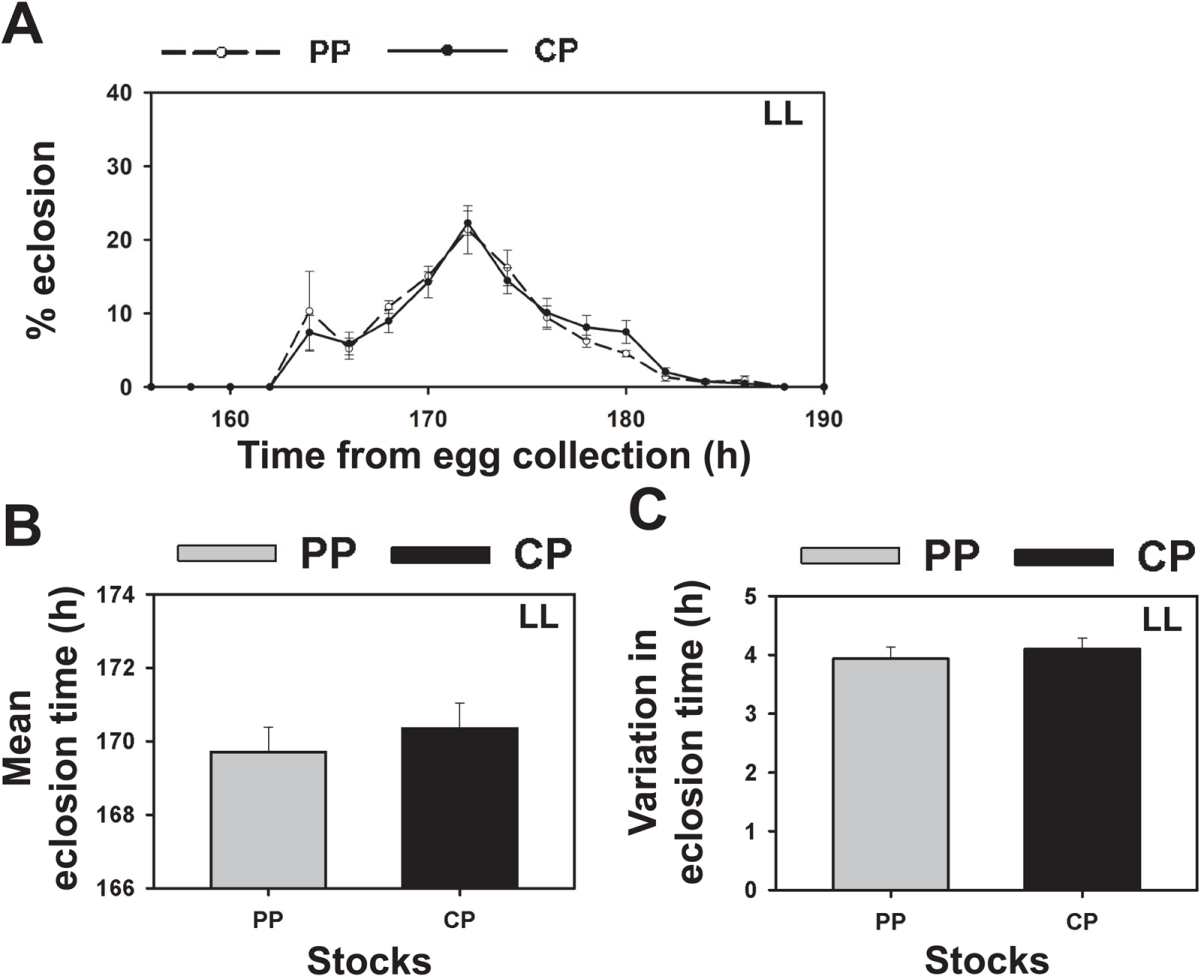
**Eclosion of selected and control populations under LL conditions.** (A) Percentage of flies eclosing in every 2 h interval measured from the time of egg collection in selected (PP, *n*=908 flies) and control (CP, *n*=982 flies) stocks under constant light (LL). (B) Mean eclosion time of selected and control stocks under LL. (C) Variation in eclosion time estimated by the standard deviation in eclosion time across all individuals of selected and control stocks under LL. Grey bars represent selected stocks and black bars represent controls. Error bars are s.e.m. across four replicate populations for each stock (*n*=4). Rest of the details same as in Fig. 1.

We also assayed the development time of selected and control stocks under constant conditions of light to assess clock-independent development in selected and control stocks (Fig. 4A–F). Under LL conditions where the *Drosophila* clock is rendered arrhythmic (Konopka et al., 1989), the eclosion profiles of selected and control stocks were virtually identical (Fig. 4A). The mean development time of control and selected stocks was ∼170 h with no significant difference between the stocks in the mean or variation in eclosion time (Fig. 4C,E; *P*>0.05; Table 1). This suggests that the differences in the eclosion profile of selected and control stocks seen in LD are due to differences in the effects of gating by the circadian clock, rather than due to differences in the developmental rate.

### Immediate response of eclosion to advance or delay of lights-on in selected and control stocks

**Fig. 5.**
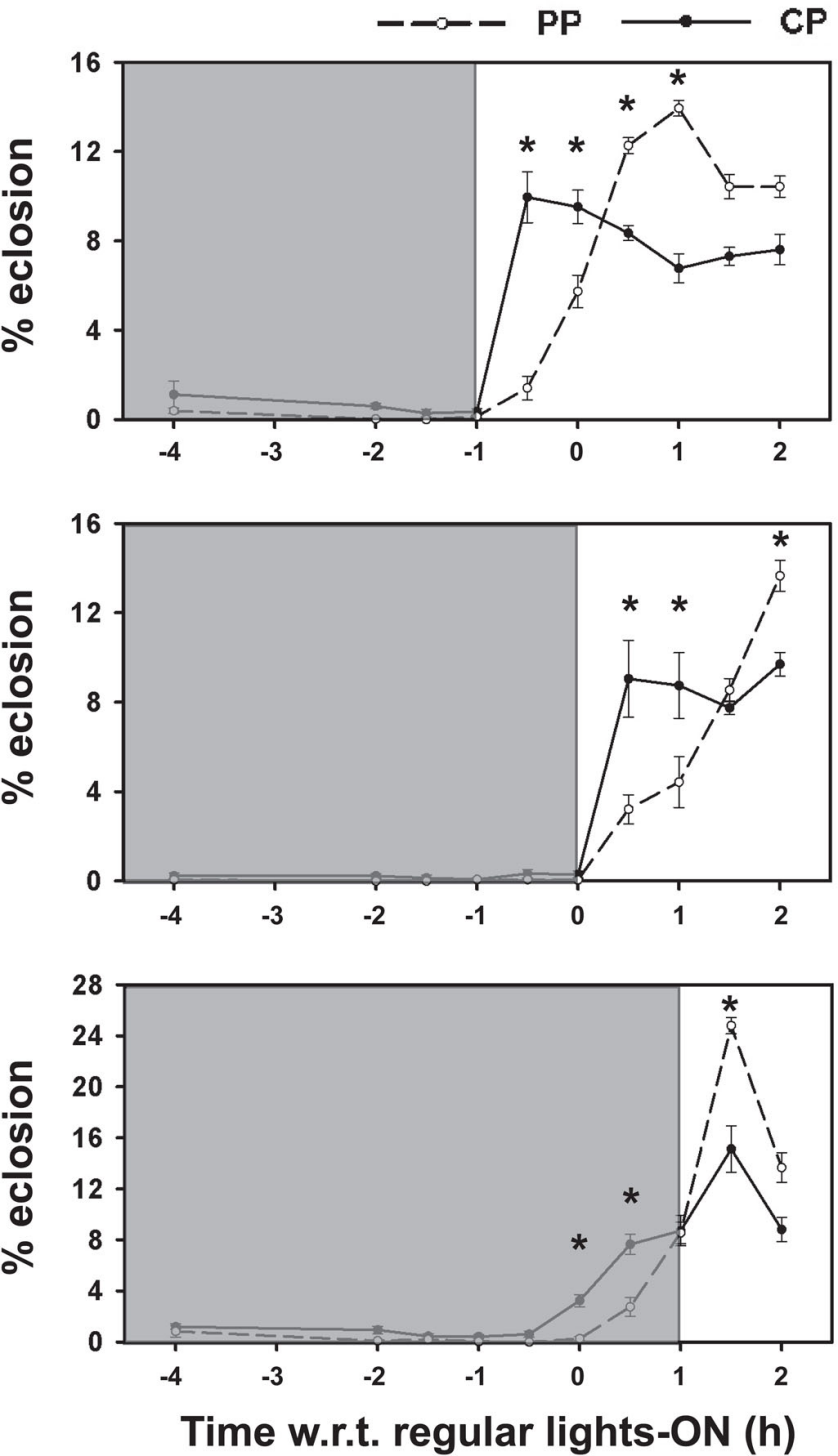
**Immediate responses of eclosion to shifted lights-on in selected and control stocks**. Percentage of eclosion in 0.5 h intervals from 2 h before regular lights-on in selected (dashed lines, total *n*=7762 flies across all three treatments) and control stocks (solid lines, total *n*=7711 flies across all three treatments) when timing of lights-on is advanced (top panel) by 1 h relative to time of regular lights-on (taken as ZT0 for all regimes), occurs at the regular time of lights-on (middle panel), or is delayed by 1 h relative to time of regular lights-on (lower panel). Lights-on occurred at the usual time during the entire development of the flies and was advanced or delayed only on the day of eclosion. Grey shading represents the time when the flies experience darkness on the day of eclosion. Error bars are s.e.m. across four replicate populations for each stock (*n*=4). *P<0.05. Rest of the details same as in Fig. 1.

Apart from innate developmental mechanisms that determine timing of eclosion, eclosion is also responsive to cues from the environment such as light. Indeed, lights-on signals may be proximate cues that convey information about time of the day and directly induce eclosion (McNabb and Truman, 2008). In order to study such immediate responses to light, we subjected the pupae to advance or delay of lights-on by 1 h on the day of eclosion, with respect to the time of regular lights-on experienced during development. We assayed the percentage of eclosion of flies from ZT22–ZT2 in bins for 0.5 h each for selected and control stocks under the advance, delay and regular LD regimes. Under normal timing of lights-on (Fig. 5, middle panel), we see high eclosion in the control stocks at the time-points of ZT0.5 and ZT1 whereas the peak of eclosion in the selected stocks lies at ZT2. While the peak of eclosion in selected stocks at ZT2 represents the endogenous peak, the eclosion seen at ZT0.5 and ZT1 in the control stocks likely represents the masking response to lights-on at ZT0. ANOVA followed by post-hoc comparisons using Tukey's HSD revealed significantly greater eclosion at ZT0.5 and ZT1 and significantly lower eclosion at ZT2 in control stocks compared to selected stocks (*P*<0.05; Fig. 5, middle panel; Table 1).

When the lights-on is advanced by 1 h (Fig. 5, top panel), the control stocks respond by increasing their eclosion at ZT23.5 and ZT0 (where ZT0 is taken as the time of regular lights-on to which the flies are entrained during development and not the time of advanced lights-on on the day of eclosion). The peak eclosion of selected stocks is also advanced due to advance in the timing of lights-on but not to the extent seen in the control stocks and the percentage of eclosion before the regular lights-on remains negligible (Fig. 5, top panel). Significantly greater eclosion at ZT23.5 and ZT0 and significantly lower eclosion at ZT0.5 and ZT1 was observed in control stocks compared to selected stocks (*P*<0.05; Fig. 5, top panel; Table 1). This indicates that flies from the selected stocks do not respond to advance in lights-on as much as the control stocks and do not eclose outside the gate of their circadian rhythm, which is entrained to the regular LD cycle experienced during development.

In contrast, when lights-on was delayed, both control and selected stocks peaked immediately after the delayed lights-on occurred. However, the peak of eclosion was greater in the selected stocks compared to the controls (Fig. 5, bottom panel) with significantly greater eclosion at ZT0 and ZT0.5 and significantly lower eclosion at ZT1.5 in control stocks compared to selected stocks (*P*<0.05; Fig. 5, bottom panel; Table 1). Overall, these results suggest that the selected stocks are less responsive to the masking effects of light compared to controls, especially when light is presented outside the eclosion gate; rather they are more tightly gated by the circadian clock.

## DISCUSSION

The evolution of accuracy of timing of circadian behaviours has been the subject of considerable interest (Pittendrigh and Daan, 1976a; Pittendrigh, 1981; Beersma et al., 1999; Sharma, 2003). While there is some evidence for evolution of clock properties as a consequence of selection for accuracy of circadian rhythms (Kannan et al., 2012a), the role of clock-independent physiological processes to enhance accuracy of behavioural output is not clear. Since it had been previously suggested that downstream output processes may contribute substantially to variation in overt rhythms (Pittendrigh and Daan, 1976b), we examined the evolution of developmental processes independent of the circadian clock that may affect eclosion timing in *Drosophila* populations selected for enhanced accuracy of eclosion rhythm.

While it is known that fruit flies do not show circadian rhythms of pupariation and pigmentation (Pittendrigh and Skopik, 1970), reduced variation between individuals in timing of transitions between these developmental stages could potentially reduce the variation in timing of eclosion. However, the selected stocks which show reduced variation in eclosion timing under LD 12:12 do not show any reduction in variation in timing of pupariation and pigmentation among individuals (Figs 1 and 2) compared to controls. Hence, the mechanisms underlying enhanced accuracy in eclosion time seen in selected stocks are likely to be restricted to advanced stages of development post pigmentation. Minor differences in the profiles of pupariation and pigmentation between selected and control stocks were observed with pupariation occurring later and pigmentation occurring earlier in selected stocks. The lack of statistical significance in these differences is probably due to the variation between replicate populations seen in the profiles and means of pupariation and pigmentation time (Fig. S1). Since development time is extremely susceptible to variation due to changes in environmental conditions, we repeated this experiment to verify these results and found similar trends of delayed pupariation time in selected stocks that were not significantly different from controls (Fig. S2). However, we did not find a trend of early pigmentation in selected stocks (Fig. S2D) as observed in Fig. 1B. Therefore, we conclude that although differences in pupariation time are consistently observed between selected and controlled stocks, these are not statistically significant and the difference disappears for pigmentation time. Thus, marginal differences in developmental rate observed at the pupariation stage would not have any bearing on overall eclosion time. Hence, eclosion (which is gated by the clock) is the only event that is more accurate in the selected stocks whereas other events such as pupariation and pigmentation (which are not clock-controlled) show similar or more variability in the selected stocks compared to controls.

While selection for early and late timing of eclosion had yielded correlated responses in pre-adult development time (Nikhil et al., 2016), we observed no significant difference in mean eclosion time between selected and control stocks in the current study (also shown in Varma et al., 2014). The lack of change in development time in these selected stocks is probably due to the selection window of eclosion being positioned close to the mean eclosion phase in our study, as opposed to the selection imposed on extreme early and extreme late phase of eclosion in the previous studies (Nikhil et al., 2016). The eclosion window in our study was so chosen to avoid directional selection for phase of eclosion while successfully selecting for greater accuracy of timing of eclosion (Fig. 2C). However, we have reported previously that selection for mean phase of eclosion in our selected stocks has resulted in evolution of shorter free-running periods of eclosion rhythms (Kannan et al., 2012a) similar to evolution of shorter and longer clock periods in response to selection for early and late phase of eclosion (Kumar et al., 2007). This may be due to the selection window of eclosion in our stocks (ZT01–02) being close to the selection window of early eclosion (ZT21–01) selected in the previous study (Kumar et al., 2007). Thus, selection for eclosion time appears to have different correlated responses for overall pre-adult development and free-running period. Moreover, the reduction in variance in eclosion time did not affect the egg-to-adult survivorship of the selected stocks (Varma et al., 2014) suggesting the lack of genetic correlations between accuracy of eclosion time and survivorship. Nevertheless, other life-history traits such as lifespan and fecundity do appear to be affected in the selected stocks with reduced lifespan and higher fecundity in selected stocks observed for flies eclosing in the selection window (Varma et al., 2014). Hence, variation in eclosion time in control stocks may be maintained due to such trade-offs between timing of eclosion and lifespan.

Although early development (pupariation and pigmentation) does not appear different between selected and control stocks, variation in proximate mechanisms leading up to eclosion independent of circadian clock control could have evolved, resulting in reduced variation in eclosion time. This role of clock-independent processes could be detected under LL conditions where the circadian clock is rendered dysfunctional (Marrus et al., 1996) and eclosion is not gated by an external cycle. Under LL conditions, selected stocks did not show differences in mean eclosion time or variation in eclosion time relative to controls (Fig. 4). These results suggest that functional circadian clocks are necessary for the lower variability in eclosion time seen in the selected stocks. Moreover, any differences in eclosion profiles under LL conditions would indicate the role of factors other than the clock in determining eclosion phenotypes of selected and control stocks. Since no such difference is observed, we conclude that no clock-independent factors are responsible for differences in eclosion profiles, consistent with other findings reported here.

In order to further substantiate evidence from these experiments that the differences in eclosion profiles between selected and control stocks are not due to interactions between developmental rate and circadian gating, we examined the eclosion profiles of both stocks under LD 12:12 while manipulating developmental rates. While developmental rate increases with temperature (Bonnier, 1926), temperature also acts as a proximate cue for eclosion behaviour. Moreover, the eclosion rhythms of the populations selected for greater accuracy have already been observed to be robust to changes in temperature when eclosion rhythms were assayed under different constant ambient temperatures of 18°C and 29°C (Kannan et al., 2012b). In this study, we manipulated developmental rates by varying larval density since development of flies is known to be significantly delayed under greater larval densities (Peters and Barbosa, 1977), with variance in development time also possibly being a function of larval crowding (Mukherjee et al., 2012). While control stocks showed greater early-morning eclosion prior to lights-on at lower larval densities, selected stocks continued to largely restrict their eclosion to the light phase (Fig. 3). If the absence of early-morning eclosion in selected stocks was simply because they have evolved a slower developmental rate, increasing the developmental rate by decreasing the larval density would be expected to yield greater eclosion prior to lights-on. Since this is not the case, we conclude that the restriction of eclosion to the light phase (or reduced eclosion before lights-on) is due to stronger gating of the circadian clock in selected stocks and not due to slower development. This is also consistent with results from previous studies on these stocks which show that manipulation of developmental rate by rearing under different ambient temperatures also does not affect the accuracy of eclosion in selected stocks (Kannan et al., 2012b). Furthermore, the phase of eclosion peak was remarkably consistent in the selected stocks across all densities compared to control stocks (Fig. 3). These results indicate that the eclosion profiles of selected stocks are robust across manipulations of larval density. Larval crowding results in delayed production of juvenile hormone esterase (JHE) and delayed release of ecdysteroids, both of which are important for timing of metamorphosis in *Tribolium freeman* (Hirashima et al., 1995). If such effects of crowding are similar in *Drosophila*, then we may conclude that changes in these hormonal variables are not critical to maintaining the enhanced accuracy of eclosion time in the selected stocks. Thus, the greater accuracy of eclosion timing in the selected stocks is largely independent of such developmental processes unconnected to the circadian clock.

Aside from developmental processes leading up to eclosion, eclosion timing can be affected by masking responses due to the direct induction of eclosion hormone release and subsequent ecdysis by light (McNabb and Truman, 2008). Such masking effects of light involve a photoreception pathway independent of circadian entrainment to LD cycles and act in addition to the gating of eclosion by the circadian clock (McNabb and Truman, 2008). Since the selected stocks had been subject to selection for a narrow window of eclosion that starts 1 h after lights-on (Kannan et al., 2012a), these stocks could potentially enhance accuracy of eclosion by evolving greater masking response to light in addition to stronger circadian gating of eclosion. However, our experiments did not reveal any enhancement of the effects of lights-on in the selected stocks compared to controls (Fig. 5). While the control stocks showed masking response to light when lights-on was advanced, such effects were minimal on the selected stocks (Fig. 5). Since the advanced lights-on was 1 h earlier than the time at which circadian gate would be opened, the relative absence of masking due to light at this time in the selected stocks suggests that circadian gating in these stocks is stronger than the direct effects of light. Although the lack of developmental readiness to eclose could also potentially be responsible for selected stocks not eclosing in response to the advancing of lights-on, we exclude this possibility due to the evidence from our pupariation and pigmentation assays and eclosion under LL that suggest no overall differences in clock-independent developmental rates between selected and control stocks. In contrast, both selected and control stocks showed masking response to light when the lights-on was delayed by 1 h (Fig. 5). Thus, the lack of masking response to advanced lights-on in the selected stocks is probably due to the greater restriction of eclosion by circadian gating rather than the direct effects of light. Therefore, these experiments also corroborate the dominant role of circadian gating in enhancing the accuracy of eclosion in the selected stocks relative to developmental processes independent of the circadian clock.

Overall, our results demonstrate that various factors independent of the circadian clock, which possibly influence timing of eclosion, such as developmental rate in early stages and under constant conditions, differences in larval densities and masking responses to light do not significantly contribute to the phenotype of enhanced accuracy in timing of eclosion observed in the selected stocks. Hence, we may conclude that the evolution of accurate eclosion rhythms of selected stocks is primarily due to stringent gating by the circadian clock. These results provide insight into the relative roles and responsivity of circadian clocks and other developmental mechanisms to selection pressures for accurate timing of eclosion in insects.

## MATERIALS AND METHODS

### Maintenance of fly populations and standardization

The study was conducted on four replicate populations each of selected (PP) and control (CP) stocks of *D.* *melanogaster*. The protocol for maintaining the populations and standardization prior to experiments is described in detail in Kannan et al. (2012a). Briefly, *Drosophila* populations that had been maintained in the laboratory for over 100 generations under LD 12:12 (light intensity ∼100 lux), constant temperature (25°C) and constant humidity (∼80%) on banana-jaggery-based food medium solidified with agar (which was used as food for all conditions unless otherwise stated, described in Kannan et al., 2012a) were the baseline populations from which PP and CP populations were derived. These populations were derived from wild-caught, outbred populations which had been maintained in the laboratory under LL conditions for over 600 generations (Sheeba et al., 1998). From the baseline populations, the four PP populations were initiated by selecting only flies that eclosed within a narrow window of 1 h, shortly after lights-on under LD 12:12 (ZT1–ZT2; where ZT0 is considered the time of lights-on). The four CP populations were also derived from these baseline populations and maintained under similar conditions as the PP populations with the exception that no selection on timing of eclosion was imposed.

Both sets of populations were maintained as large, outbred populations with about 1200 breeding adults in plexiglass cages (25 cm×20 cm×15 cm) with roughly equal males and females on a 21-day discrete generation cycle. Prior to egg collection, petriplates with banana-jaggery medium coated with yeast paste (yeast plate to induce egg-laying) were provided to the fly populations. Three days later, the yeast plate was withdrawn and replaced by a fresh food plate where vertical cuts were made on the medium and the edges of the food exposed so as to increase area of vertical surfaces available for egg laying (cut-plate). Flies were allowed to lay eggs for 2–3 h before the cut-plate was withdrawn and eggs were collected and transferred into glass vials (20 cm height×2.5 cm diameter) containing ∼10 ml of food. The eggs were dispensed at high densities of roughly 300 eggs per vial to facilitate eclosion over several days. Adult flies eclosing between the 9th and 12th days after egg collection were collected into fresh plexiglass cages to constitute the next generation. While all such flies were collected in CP populations, only those that eclosed in the selection window were chosen to form the breeding population of the next generation in case of PP populations. The selection was carried out on four successive days from eggs that were collected within a 2 h window (flies that eclose across the four days are from a single developmental cohort) in order to avoid indirect selection for development time. The assays described in this chapter have been conducted on populations that have been subject to over 120 generations of selection. The selected (PP_1–4_) populations and control (CP_1–4_) populations will be collectively referred to as selected and control stocks.

All assays were conducted on standardized flies from selected and control populations which were subjected to a common rearing protocol for one generation where selection pressure was relaxed. The populations which underwent such a generation of common rearing are referred to as standardized populations and the progeny from these populations constituted the sample flies for the assays. All assays were conducted under ambient temperature of 25°C and constant humidity (∼80%) with light intensity ∼100 lux maintained throughout the LL regime and the light phase of LD 12:12.

### Pupariation, pigmentation and eclosion assays

Standardized selected and control populations were given yeast plates for 2 days prior to the day of egg-collection to increase egg-laying. On the day of egg-collection, a cut-plate was given to all populations at ZT0 (time of lights-on) which was discarded and replaced by another cut-plate after 2 h. The second cut-plate was withdrawn 2 h later and individual eggs were transferred on to agar pieces. Agar pieces with low density of exactly 30 eggs each were then transferred to 10 vials (*n*=300 flies per population, *n*=1200 each for selected and control stocks) with ∼10 ml of food for each replicate population and maintained under LD 12:12. Low density of eggs (30 eggs/vial) for assay of development time was chosen so as to measure the overall development time of all individuals of a vial in contrast to the maintenance protocol where high density (300 eggs/vial) was used, in order to obtain multiple cycles of eclosion to select for accurate daily timing of eclosion, rather than a particular speed of development. Duration of pupariation and wing pigmentation was estimated by counting the number of pupae formed or pigmented every 2 h. Pupariation was identified by the formation of two spiracles on the head and pigmentation was recorded when the colour of wings on the sides of the pupae changed from white to grayish or black (Qiu and Hardin, 1996). Finally, the number of flies eclosing every 2 h was recorded to estimate the timing of eclosion. All durations of development were estimated as time from egg collection since all eggs in the experiment were collected in a 2 h window similar to the maintenance conditions. Hence, the pupariation and pigmentation timing corresponds to the eclosion timing following the developmental trajectory of the same vials. This enables the evaluation of inter-individual variation in pupation and pigmentation time for a 2 h developmental cohort age-matched at the egg stage and facilitates comparisons of the evolution of inter-individual variation in eclosion time due to artificial selection, to variation in timing of early developmental events. All handling and counting procedures in the dark phase of the LD cycle were performed in the presence of dim far-red light (>600 nm).

### Eclosion at different larval densities

Patterns of eclosion were determined by collecting flies that eclosed from stocks raised at different larval densities every 2 h under LD 12:12. For these assays, eggs were collected from the standardized populations and transferred into vials with ∼10 ml of food at approximately 75, 150, 225 and 300 eggs per vial. Five such vials per replicate population for each density were maintained under LD 12:12. These vials were monitored till the onset of eclosion and thereafter the number of flies eclosing was counted every 2 h for four consecutive days.

### Development time under constant conditions

The development time assay under LL was carried out using a similar protocol as that of the pupariation, pigmentation and eclosion assay with 30 eggs/vial as described above with the exception that the vials with eggs were placed under LL instead of LD 12:12 and only eclosing flies were counted. Vials were examined for the start of eclosion after which eclosing flies were counted every 2 h.

### Immediate responses to light of eclosion

Eclosion assays were conducted with 15 vials for each replicate population with eggs collected from standardized selected and control stocks at a density of ∼300 eggs per vial under three different regimes. Vials of all three regimes were placed under LD 12:12 till the day before eclosion started. While one set of vials continued to remain in LD 12:12, two other sets of vials were removed from the LD 12:12 cubicle after the lights-off on the previous day. One set of vials was exposed to a regime wherein lights-on occurred 1 h prior to the time of lights-on in the regular LD 12:12 under which they were maintained until then, while the other set of vials was exposed to lights-on occurring 1 h after the time of regular lights-on. Eclosion was monitored every half an hour from ZT22–ZT2 and subsequently, the total number of flies eclosed at ZT20 were counted to normalize the eclosion profile by the total number of flies eclosing in the day. Eclosion under respective regimes was monitored over 3 days with different sets of vials used every day.

### Statistical analyses

Pupariation profiles with 2-h resolution were estimated for each vial by calculating the percentage of flies pupating within each 2-h window (each window is denoted as a time-point where time-point is represented by time from egg collection) for a single vial and averaged across 10 vials for each replicate population. These population mean profiles were then averaged across four replicate populations for selected and control stocks and plotted with standard error of mean across the four populations as error bars. Mean pupariation time for each replicate population was also calculated and these population means were used as replicate values for one-way ANOVA with ‘stock’ as fixed factor. Variation in pupariation time was also estimated for each replicate population by calculating the standard deviation in pupariation time across all individuals and was analyzed similar to the mean. Similarly, pigmentation profiles, mean pigmentation time and variation in pigmentation time were estimated for selected and control stocks and compared. Eclosion profiles were calculated across 2 days and plotted similar to pupariation and pigmentation profiles. Mean eclosion time was calculated for all flies eclosing over 2 days and compared using ANOVA with ‘stock’ as a fixed factor. However, variation in eclosion time was calculated only for the second day of eclosion since there were very few numbers of flies eclosing on the first day. Eclosion profiles, mean eclosion time and variation in eclosion time under LL conditions were analyzed similar to pupariation and pigmentation time.

For eclosion assays with different larval densities, eclosion profiles were calculated as percentage of eclosion in each 2 h interval normalized by total number of flies emerging in a day for each vial and averaged across days 8–11. Only days in which at least 25 flies eclosed were considered for analysis from each vial and therefore, at lower densities (75 and 150 eggs/vial) mostly days 8 and 9 were used, while days 9, 10 and 11 were primarily used for higher densities (225 and 300 eggs/vial). After vial-wise averages were calculated across days, the eclosion profiles were then averaged across five vials for each replicate population at each larval density. These population mean profiles were further averaged across four replicate populations to obtain average profiles for selected and control stocks and plotted with SEM across replicate populations as error bars. Further, percentage of eclosion in the 6-h window prior to lights-on (ZT18–ZT0) was calculated as this part of the profile was the most variable across densities. ANOVA was performed on percentage of eclosion before lights-on with ‘density’ and ‘stock’ as fixed factors. The phase of peak of eclosion was also recorded as the time at which maximum eclosion was observed on a given day for a vial. This phase of eclosion peak was averaged across days and across all vials within a population and subsequently averaged across four replicate populations for each stock and plotted. ANOVA with ‘density’ and stock’ as fixed factors was performed on phase of eclosion peak.

To study the direct effect of light on eclosion, 0.5 h eclosion profiles were calculated from ZT22–ZT2. The percentage of eclosion in each 0.5 h interval was calculated as a fraction of the total eclosion in a day for a particular vial and averaged across all vials within a population and subsequently, averaged across the four populations and plotted. ANOVA with ‘stock’ and ‘time-point’ (which is the time of the day depicted in 2-h windows as ZT0, ZT2, etc.) as fixed factors was performed separately for each regime.

Data were tested for normality using Kolmogorov–Smirnov test. All post-hoc comparisons were done using Tukey's Honest Significant Difference. All statistical analyses were performed on STATISTICA 7.0 and differences were considered statistically significant at *P*<0.05.

## Supplementary Material

Supplementary information
