# Supplementary material for: Accuracy of fruit-fly eclosion rhythms evolves by strengthening circadian gating rather than developmental fine-tuning
Source: Biol Open. 2019 Aug 15;8(8):bio042176. doi: 10.1242/bio.042176 (PMC6737981; doi:10.1242/bio.042176)
Supplement: Supplementary information [file biolopen-8-042176-s1.pdf]

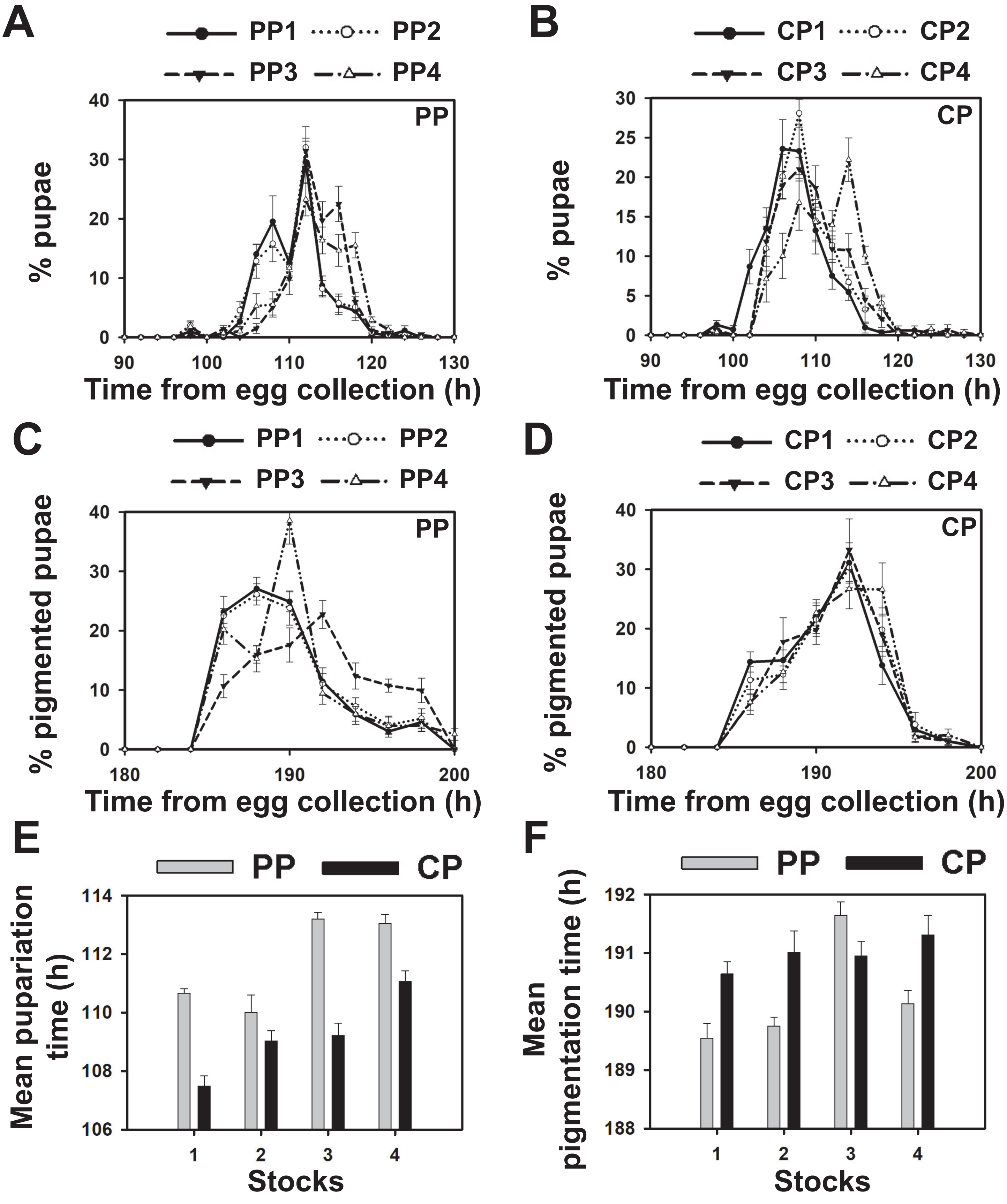

Varma et al. Figure S1. Pupariation and Pigmentation of four replicate selected and control populations under LD 12:12. Percentage of larvae that pupated in every 2 hour interval measured from the time of egg collection in A) selected (PP1, PP2, PP3, PP4 represented by solid, dotted, dashed and semi-dashed lines respectively) and B) control (CP1, CP2, CP3 and CP4 represented similarly as PP populations) stocks and proportion of pupae that pigmented every 2 hours in C) selected and D) control stocks under LD 12:12. E) Mean pupariation time of four replicate populations of selected and control stocks under LD 12:12. F) Mean pigmentation time of four replicate populations of selected and control stocks under LD 12:12. All values are estimated from single vials containing 30 flies and subsequently averaged across 10 vials for each replicate population. Bar graphs and line plots represent mean values and error bars are SEM across ten vials for each replicate populations (n = 10).

Biology Open • Supplementary information

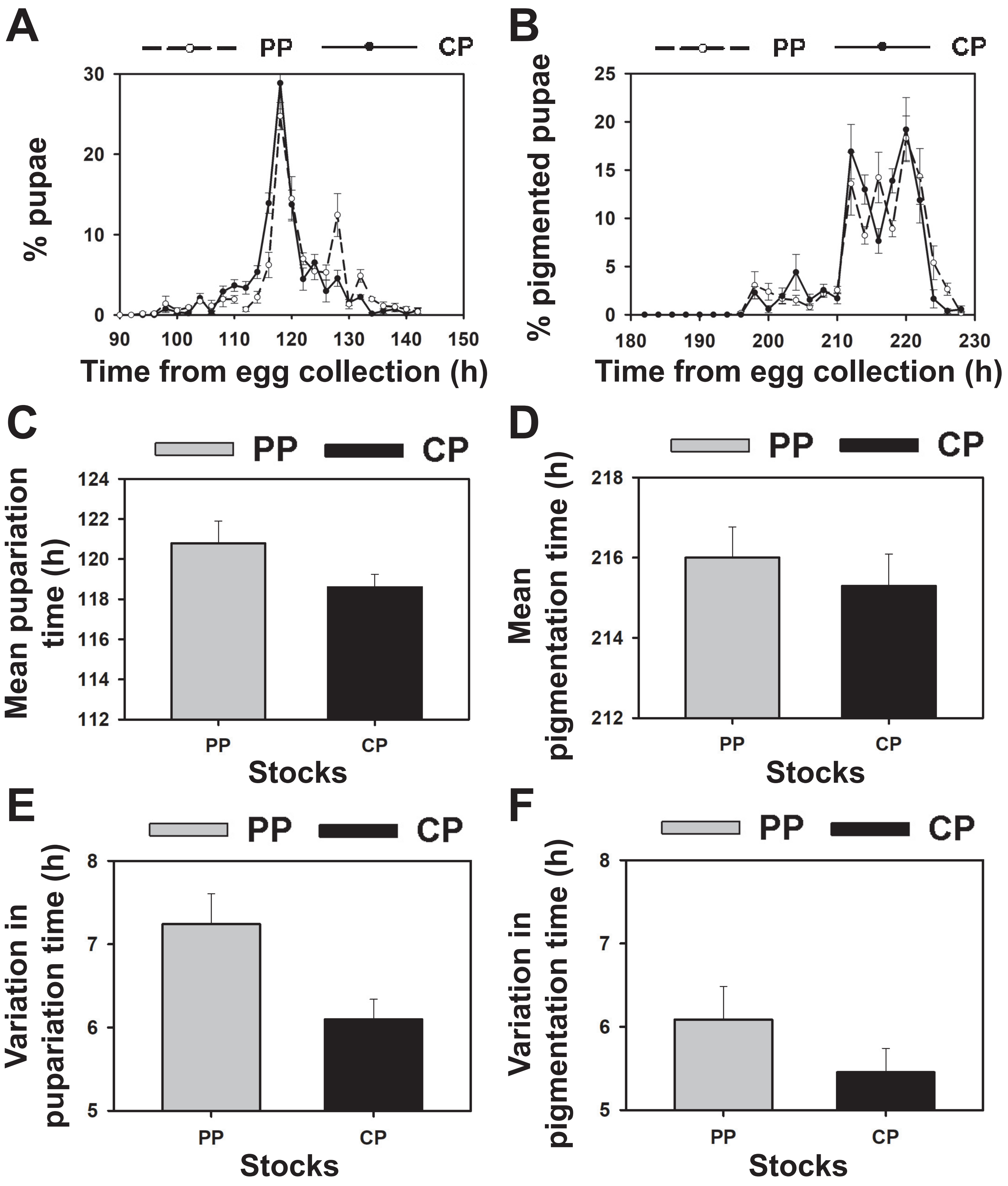

Varma et al. Figure S2. Pupariation and Pigmentation in selected and control populations under LD 12:12. A) Percentage of larvae that pupated in every 2 hour interval measured from the time of egg collection in selected (PP) and control (CP) stocks under LD 12:12. B) Percentage of pupae from selected and control stocks that showed wing pigmentation in every 2 hour interval under LD 12:12. C) Mean pupariation time of selected and control stocks under LD 12:12. D) Mean pigmentation time of selected and control stocks under LD 12:12. E) Variation in pupariation time estimated by the standard deviation in pupariation time across all individuals of selected and control stocks. F) Variation in pigmentation time across all individuals of selected stocks and control stocks. All values are estimated from single vials containing 30 flies and subsequently averaged across 10 vials for each replicate population. Bar graphs and line plots represent mean values and error bars are SEM across four replicate populations for each stock (n = 4).

Biology Open • Supplementary information
